# PLIC: protein–ligand interaction clusters

**DOI:** 10.1093/database/bau029

**Published:** 2014-04-23

**Authors:** Praveen Anand, Deepesh Nagarajan, Sumanta Mukherjee, Nagasuma Chandra

**Affiliations:** ^1^Department of Biochemistry, Indian Institute of Science, Bangalore 560012, Karnataka, India and ^2^IISc Mathematics Initiative, Indian Institute of Science, Banglaore 560012, Karnataka, India

## Abstract

Most of the biological processes are governed through specific protein–ligand interactions. Discerning different components that contribute toward a favorable protein– ligand interaction could contribute significantly toward better understanding protein function, rationalizing drug design and obtaining design principles for protein engineering. The Protein Data Bank (PDB) currently hosts the structure of ∼68 000 protein–ligand complexes. Although several databases exist that classify proteins according to sequence and structure, a mere handful of them annotate and classify protein–ligand interactions and provide information on different attributes of molecular recognition. In this study, an exhaustive comparison of all the biologically relevant ligand-binding sites (84 846 sites) has been conducted using PocketMatch: a rapid, parallel, in-house algorithm. PocketMatch quantifies the similarity between binding sites based on structural descriptors and residue attributes. A similarity network was constructed using binding sites whose PocketMatch scores exceeded a high similarity threshold (0.80). The binding site similarity network was clustered into discrete sets of similar sites using the Markov clustering (MCL) algorithm. Furthermore, various computational tools have been used to study different attributes of interactions within the individual clusters. The attributes can be roughly divided into (i) binding site characteristics including pocket shape, nature of residues and interaction profiles with different kinds of atomic probes, (ii) atomic contacts consisting of various types of polar, hydrophobic and aromatic contacts along with binding site water molecules that could play crucial roles in protein–ligand interactions and (iii) binding energetics involved in interactions derived from scoring functions developed for docking. For each ligand-binding site in each protein in the PDB, site similarity information, clusters they belong to and description of site attributes are provided as a relational database—protein–ligand interaction clusters (PLIC).

**Database URL**: http://proline.biochem.iisc.ernet.in/PLIC

## Introduction

Protein–ligand interactions play a vital role in all biological processes ranging from metabolic enzyme catalysis to regulation of complex signaling cascades. Knowledge on molecular details of these interactions is crucial for complete understanding of the biological system. The large-scale structural information available on protein–ligand complexes has led to the development of various computational approaches that analyze protein–ligand interactions in terms of different attributes such as atomic contacts, binding energetics and shape recognition features. It has long been realized that multiple factors or attributes collectively contribute to favorable protein–ligand interactions. These attributes can be roughly divided into binding site properties of the protein, protein–ligand atomic contacts and different components of binding energetics involved in the interaction.

Several protein–ligand databases such as BioLiP (1), Credo (2), Possum (3), Pocketome (4), Relibase (5), scPDB (6), Probis (7) and PLI (8) are available in literature. Each of them reports a unique type of information about protein–ligand interactions. *Probis* analyzes similarity at the substructure level across different protein structures along with conservation scores, whereas *Possum* reports ligand-binding site similarities. Credo reports the similarity of binding site shapes using the FuzCav algorithm (9). Although most of these tools (10, 11) detect similarities in interactions using their own scoring scheme, none of them reports details of the underlying attributes such as binding site shape, protein–ligand contacts, energetics and variation of these attributes across similar protein–ligand interactions. Here we present a database providing the Protein Data Bank (PDB)-scale information of all similar binding sites for each protein–ligand complex. In-house tools, PocketMatch (12) and PocketAlign (13), have been used to obtain clusters of similar binding sites from the PDB. The PocketMatch algorithm represents a binding site in a frame-invariant manner by considering both shape and chemical nature of the amino acid. A pair of binding sites is then compared based on alignment of 90 lists of sorted distances obtained for each of the sites. A comprehensive validation and sensitivity analysis (12) has been performed for this algorithm on different data sets (14). An all-pair comparison of binding sites has been performed using the PocketMatch algorithm, and a binding site similarity network (15--17) has been constructed using the reported similarity score. The clusters are then extracted from the network using the Markov clustering (MCL) algorithm (18). The structural alignment of binding sites for each cluster is then obtained using another in-house algorithm—PocketAlign (13). Along with these, various other widely used computational tools including fPocket (19), Autodock (20) and EasyMIFs (21) have been used to study the other attributes of these interactions across the clusters of similar binding sites.

## PLIC workflow

**Figure 1.**
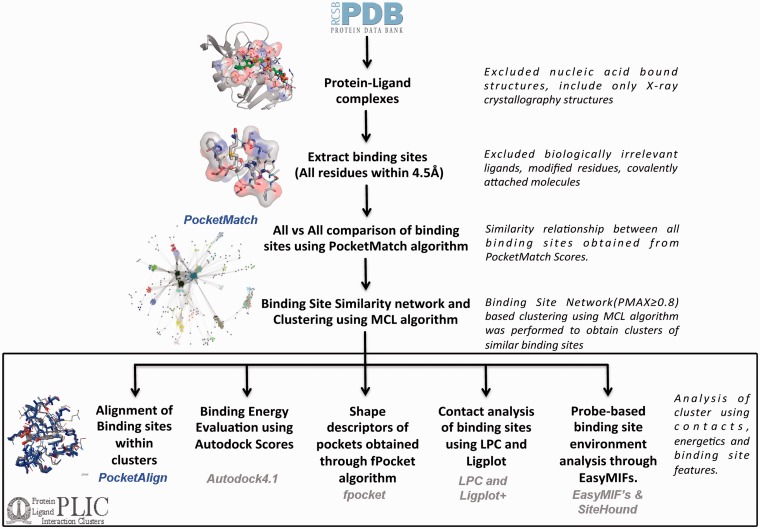
PLIC database workflow. The flowchart illustrates the different steps involved in the construction of the PLIC database. All the protein–ligand complexes are downloaded from the PDB, and binding sites (comprising all the residues that are within 4.5 Å of any ligand atom) are extracted. Only the biologically relevant ligands are selected that resulted in 84 846 binding sites. An exhaustive all-versus-all comparison of these 84 846 binding sites is performed using PocketMatch, and a binding site similarity network is constructed at a PMAX cutoff of 0.8. Network-based clustering of binding sites is performed using the MCL algorithm to obtain clusters of similar binding sites. All the different attributes that are calculated for the interactions within the clusters along with computational tools that were used to derive them are mentioned in the box.

All the protein–ligand complexes were derived from the PDB (as of 30 October 2012). To keep up with the rapid growth of the PDB, the protein–ligand interaction clusters (PLIC) database has been updated to display related entries for the 25th February 2014 version of the PDB. Protein–nucleic acid complexes were filtered out in the very first step through the advanced query option in the PDB. Metal ions, covalently bound ligands and crystallization agents were excluded. Modified residues were also filtered out, as these would be represented as heteroatom (HETATM) in the PDB file. Altogether there were ∼311 ligands, 67 metal ions and 485 modified residues that were excluded. The complete list can be accessed through the web site at: http://proline.biochem.iisc.ernet.in/PLIC/excluded_ligands.txt. A distance threshold of 4.5 Å was used to extract the binding sites from the remaining 30 956 protein–ligand complexes. The binding site can be defined as a set of residues belonging to the protein that lies within the zone of 4.5 Å from any atom of the corresponding ligand. Although no threshold was adopted for the ligand size, peptides were automatically excluded (because the unwanted ligand list also included all 20 amino acids), and a threshold of five residues in the binding site was chosen to exclude non-specific interactions. Around 84 846 sites were extracted from 30 956 protein–ligand complexes. The majority of the binding sites had 15–20 residues in them (Supplementary Figure S1). An exhaustive all-versus-all comparison of these 84 846 binding sites was performed using the PocketMatch algorithm. The algorithm reports two scores—PMIN, which estimates the local similarity score, and PMAX, which estimates a global similarity score of a given pair of sites. Statistical significance of both the scores is reported through *P*-values. A PMAX score of 0.40 is known to be significant through a large-scale comparison of pockets belonging to proteins of the same fold (12). Scores >0.4 increasingly reflect higher extents of similarity with an identical pair of sites exhibiting a PMAX score of 1. A default PMAX cutoff of 0.80, indicating high similarity, was used to construct a binding site similarity network. Each node in the network represents a binding site, and an edge is drawn between them if the PMAX score of the corresponding pairs is ≥0.80 (Supplementary Figure S2). The MCL algorithm (18) is then used over the binding site network to derive clusters containing similar binding sites. Around 10 858 binding site clusters were obtained (Supplementary Figure S3). The node with the highest degree within the cluster was selected as a representative site and used as a reference to align all other sites in the cluster onto it. The entire workflow has been depicted in Figure 1. The decision to make the threshold more stringent is based on the goal of identifying only high-confidence similarity pairs, thus minimizing false-positive findings. This is, perhaps, at the cost of missing out some important pocket pairs at a lower PMAX cutoff, which implies that there could be some false-negative findings that may be missed out. Hence, the clustering was also performed on the binding site network constructed using different PMAX cutoff values at 0.6, 0.7 and 0.9.

The PMAX score reported by the PocketMatch algorithm here reflects the similarity of the binding site environment in terms of chemically similar residues present at topologically equivalent positions around the ligand. One way to validate the clusters obtained through this similarity relationship is by looking into the chemical similarity of the ligands that these binding sites enclose. Tanimoto chemical similarity was evaluated using Open Babel toolbox (22) for all the ligands present in clusters obtained at different PMAX cutoffs. In general, the number of clusters obtained increased with the PMAX cutoff, and most of these clusters were observed to contain only one type of ligand (Supplementary Table S1). The average Tanimoto chemical similarity scores also improved with the higher PMAX cutoff (Supplementary Figure S4). However, at a high PMAX cutoff of 0.9, the number of clusters obtained surpassed (11 886 clusters) the total number of unique ligands (11 042 unique ligand codes), indicating high sensitivity because similar sites are split into different clusters and hence are unhelpful in determining relationships across different sites. Thus, a default PMAX score cutoff of 0.8 was retained for the analyses. The option of choosing a different similarity cutoff and retrieving the corresponding clustering results has also been provided.

Different attributes, as mentioned above, were derived for each of the protein–ligand interactions within the clusters. Around 14 general binding site descriptors are listed for each of the pockets, with the majority of them derived from fpocket (19). The attributes capturing the binding energetics involve Autodock 4.1 scores and different energetic contributions consisting of electrostatic, hydrogen bond, van der Waals, desolvation and torsional score components. The binding site environment was further analyzed in terms of interaction profiles using EasyMIFs and Sitehound. The binding site environment (35-Å^3^ grid from the centroid of ligand) was scanned with a methyl probe, hydroxyl probe, phosphate probe and an aromatic carbon probe. The favorable interaction zones of these probes can be derived in the form of clusters depending on their interaction energy in the protein environment using Sitehound (21). All the atomic contacts of proteins with the ligands are captured using Ligand-Protein Contacts (LPC) software (23). Residues that interact with ligand atoms through hydrogen bonds, aromatic interactions and hydrophobic interactions are also added to the database. The information on variation of all these attributes within the clusters is plotted in the form of box plots. Additionally, the CATH superfamilies are also tagged with each of the binding sites, facilitating correlation of the CATH superfamily type with specific ligand types. Table 1 lists all the types of protein–ligand interaction attributes that one can obtain from the database.

**Table 1.** Attributes of interaction

Attribute type	Attribute name	Computational tools used
Binding site descriptors	Pocket volume	fpocket LPC
	Number of alpha spheres	
	Mean alpha sphere radius	
	Proportion of apolar alpha spheres	
	Mean local hydrophobic density	
	Hydrophobicity scores	
	Volume score	
	Charge score	
	Proportion of polar atoms	
	Alpha sphere density	
	Max. distance between COM and alpha sphere	
	Max. theoretical shape complementarity	
	Observed shape complementarity	
	Normalized shape complementarity	
Binding site environment and binding energetics	Autodock score	Autodock4.1 EasyMIFs SiteHound
	Electrostatic score	
	Hydrogen bond score	
	van der Waal score	
	Desolvation score	
	Torsional score	
	Average methyl probe (CMET) interaction energy	
	Total CMET interaction energy	
	Total CMET interaction grids	
	Total CMET interaction clusters	
	Average phosphate probe (OP) interaction energy	
	Total OP interaction energy	
	Total OP interaction grids	
	Total OP interaction clusters	
	Average hydroxy probe (OA) interaction energy	
	Total OA interaction energy	
	Total OA interaction grids	
	Total OA interaction clusters	
	Average aromatic probe (CR1) interaction energy	
	Total CR1 interaction energy	
	Total CR1 interaction grids	
	Total CR1 interaction clusters	
Ligand–protein contacts	Hydrogen bonds	Ligplot+ LPC
	Aromatic	
	Hydrophobic	
	Destabilizing	
	Donor water molecules at the binding site	
	Acceptor water molecules at the binding site	

All the attributes that are calculated for each of the interactions present in the database along with the computational tools used to derive them have been listed in this table.

## Database

**Figure 2.**
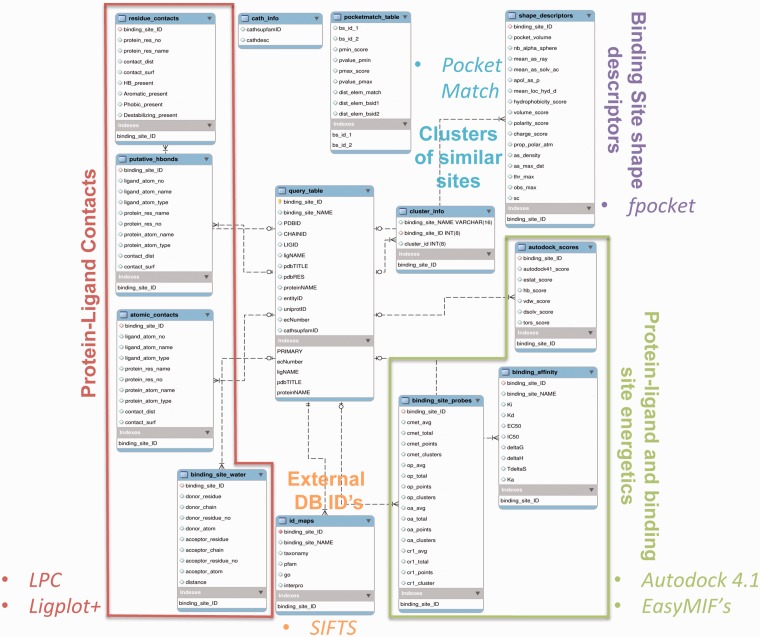
The EER of the PLIC database. The EER of different data types in PLIC is shown. The database consists of 13 tables, and the relationship between these tables is depicted here. The logical partition indicating the type of information is highlighted and labeled with different colors.

Information on all 84 846 protein–ligand interactions, along with binding site similarity scores, cluster information and the values of various attributes, has been stored in the MySQL database. Figure 2 summarizes the enhanced entity relationship (EER) between different tables of the database, and the logical partition specifies the type of data represented in the table. The database can be queried in multiple ways by using any of the PDBID (24) (RCSB identifier), LIGID (ligand identifier, three-letter HETATM code), UniprotID (25) (Uniprot database identifier), EC number (enzyme commission number), CathID (26) (CATH superfamily ID) identifiers. The query can also be performed through protein and ligand names. Multiple queries can also be combined to filter out a specific set of information. For example, if one is interested in the EC number 1.5.1.3, dihydrofolate reductase enzyme, and retrieving all the information from the database where this EC number is associated with a specific inhibitor—MTX (methotrexate)—then the user can select the checkboxes for both the EC number and LIGID to enter the EC number 1.5.1.3 and LIGID—MTX, simultaneously. The entries in the database have also been mapped to IDs from different databases to enhance the browsing capability. The external databases linked include—‘Interpro’ (27), ‘Pfam’ (28), ‘Gene Ontology’ (29) and ‘NCBI taxonomy’ (30). The mapping between different IDs has been obtained from the SIFTS database (31). The binding affinity data have also been derived from the PDB and are included as one of the browse capabilities. These affinity values in PDB are compiled from other databases, including PDBbind (14), BindingMOAD (32) and BindingDB (33). A separate dedicated browsing interface has been provided on the web-server to perform specific queries involving the information from the aforementioned databases.

All the entries present in the database are made unique through their binding site name. The binding site name consists of four fields concatenated together with an underscore separator ‘_’. The first field is the PDBID of the protein in the Research Collaboratory for Structural Bioinformatics (RCSB) database. The second field represents the unique three-letter code for the ligand entity. The third and the fourth fields represent the chainID and the residue number of the ligand entity, respectively, as present in the PDB file. An instance of MTX bound to dihydrofolate reductase can be represented as ‘1dhi_MTX_A_161’ in our database. ‘1dhi’ stands for one of the PDB codes of the dihydrofolate reductase protein deposited in PDB, MTX stands for HETATM code of MTX ligand and ‘A’ and ‘161’ stand for the chainID and the residue number of ligand, respectively.

A Web interface was created for the database through php (http://php.net). The interactions between the protein–ligand and the alignment of sites belonging to a cluster with the reference are displayed using a *Jmol* applet (http://jmol.sourceforge.net/). The graphical results of various analyses performed on the cluster are displayed through *highcharts* (http://www.highcharts.com/) java plug-in. *jQuery* (http://jquery.com/) plug-ins have been used appropriately to display the results of the database query tables. The representational state transfer design architecture has been used to design the application programming interface for the PLIC database. All the tables in the PLIC database can be programmatically queried to get the results in the JavaScript Object Notation (JSON) format. A detailed help section has been added that explains the details of the query along with the snapshots explaining the results.

## Database statistics

**Figure 3.**
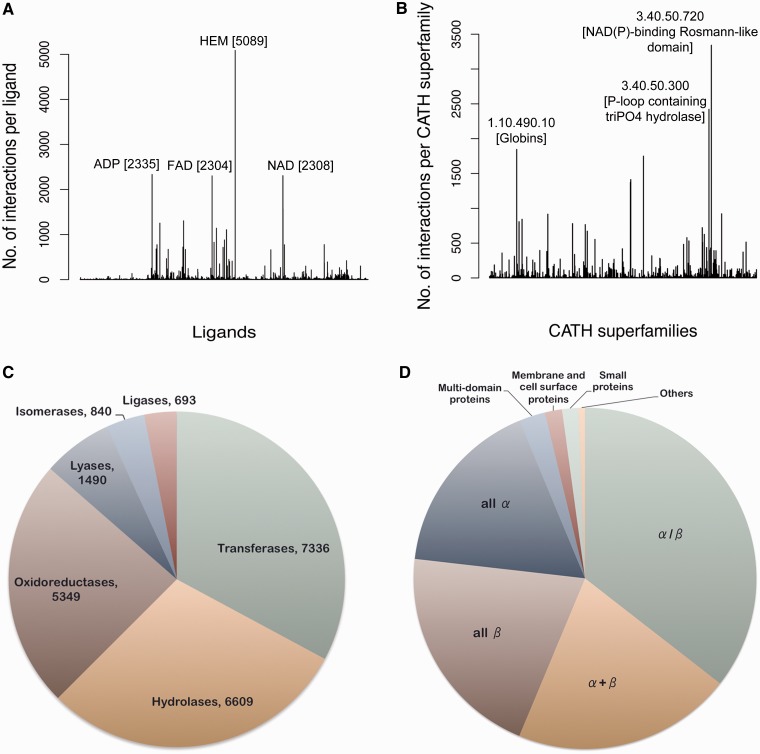
Database statistics. (**A**) The frequency of different ligand-binding sites present in the database is represented in the form of a histogram. The most populated ligands are labeled along with their frequencies. (**B**) The number of interactions present per CATH superfamily is depicted in the form of a histogram. The CATH superfamilies associated with most number of ligands are labeled. The pie charts depict the distribution of different (**C**) enzyme classes and (**D**) SCOP classes present in the database.

Currently, the database consists of 84 846 protein–ligand interactions from 30 956 PDB entries. The total number of unique ligands totals 11 042. The most abundantly (5089 sites) present ligand molecule is HEM, protoporphyrin IX containing iron (Fe), known to interact with proteins belonging to as many as 36 different CATH (26) superfamilies. Figure 3A shows the frequency distribution of different ligands present in the data set. Around 62% of the entries in the data set have a CATH superfamily associated with it. The most abundant of the CATH superfamilies is 3.40.50.720, Nicotinamide adenine dinucleotide phosphate (NAD(P))-binding Rossmann-like domain (3342 occurrences), having an association with 240 different ligand codes. Figure 3B depicts the CATH superfamily distribution. Around 60% of the proteins in the database have an EC number associated with them. The most abundant class of enzyme in the database appears to be transferases, with nitric oxide synthase having EC number 1.14.13.39 being most frequent. Figure 3C shows the distribution of different enzyme classes in the database. Structural Classification of Proteins (SCOP) is another database like CATH that classifies the protein structure systematically depending on the secondary structure composition and connections. Information about the SCOP classification of proteins present in the database can also be obtained from this analysis. The most abundant SCOP class is the α/β category followed by the α + β category (Figure 3D).

## Example query

Output generated through a standard query in the database will be discussed here by taking MTX as an example. MTX is a common drug used in the treatment of arthritis and various rheumatic conditions (34). It is also used in the treatment of gastric cancer (35) and a host of other conditions (36). The identification of conserved structural and functional characteristics of MTX binding sites could aid studies into the origin and treatment of the conditions previously mentioned.

**Figure 4.**
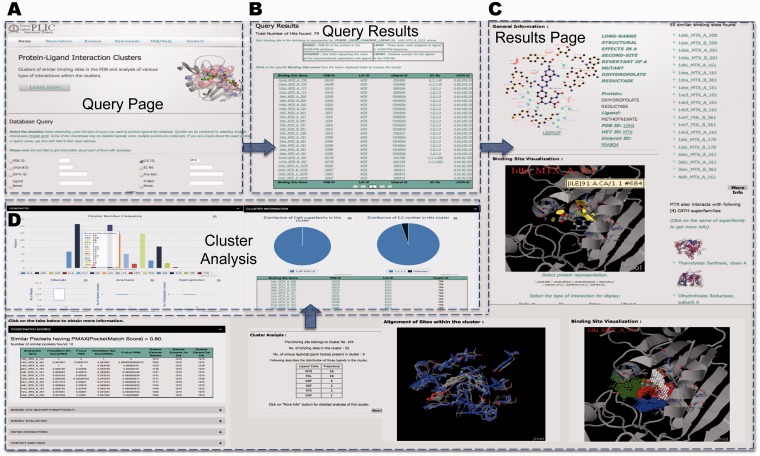
PLIC database server. (**A**) Snapshot of the query page for the PLIC database. (**B**) The page displaying the results of the query in the tabular form containing information about the name of the binding site, protein, ligand, UniprotID, EC number and CATH superfamily ID. (**C**) The results page displayed after a specific binding site name is clicked. The results page consists of Jmol plug-in for visualization of interactions, clusters indicating high-energy interaction zones for different probes, alignment of binding sites within the cluster, similar sites with PocketMatch scores, cluster information and various attributes associated with the interaction. (**D**) Barplot illustrating the distribution of various residues within the binding site environment of the cluster and box plots indicating the variations observed in different attributes of interactions within the cluster are displayed on the cluster analysis page.

A simple query of ligand ID MTX (three-letter HETATM code for methotrexate) reveals that ∼79 protein–ligand interactions involve MTX (Figure 4A). The query results are displayed in the tabular form with the information about the binding site name, protein PDB IDs with corresponding Uniprot entries with information on EC number and the CATH superfamily ID (Figure 4B). The query reveals that MTX interacts with proteins belonging to different enzyme classes—thymidylate synthase (EC number 2.1.1.45), dihydrofolate reductase (1.5.1.3) and pteridine reductase (1.1.1.253). The specific details of the interaction can be obtained by clicking on the binding site name (details of the nomenclature are described in the Database section and are also explained on the query page).

The results page shows the pictorial representation of the interaction as displayed by Ligplot (37) (Figure 4). Results for 1dhi_MTX_A_161 reveal that there are ∼55 sites that are similar to this particular binding site falling into cluster number 104. All the sites that are similar (PocketMatch PMAX >0.8) to this binding site (in this case, 12 of them), along with similarity scores, are displayed. The unique CATH superfamilies listed reveal that MTX also interacts with immunoglobulins (antibodies engineered to bind to MTX). The other ligands that fall into this cluster include the natural substrates folate and dihydrofolate, as well as different inhibitors—diazatetrahydrofolic acid, deazafolic acid and 5-formlyl tetrahydrofolate.

The binding energetic interaction analysis through Autodock 4.1 scores reveals that a major contribution toward favorable interaction energy is through van der Waals contacts. Binding site environment probe analysis reveals that the highest number of clusters (10 clusters) is obtained for the hydroxyl probe, followed by a methyl probe (7 clusters), phosphate probe (6 clusters) and the aromatic probe (5 clusters). This gives us an idea about the nature of the binding site that could be an important input for 3D pharmacophore design. Most of the interacting grid points for the clusters in the binding site environment are also in the same order as mentioned above (cutoff of −8 used for all the probes). The interaction zones of the clusters for each of the probes can be visualized in the binding site environment.

The contact analyses reveals that there are 11 potential hydrogen bond contacts formed between the protein and ligand in 1dhi_MTX_A_161, followed by 6 hydrophobic contacts and only 1 aromatic contact contributed by Phe31. The cluster level analyses reveal that the number of these contacts is conserved across all the sites within the cluster. All the other attributes that are mentioned in Table 1 can also be obtained readily. The cluster-level analyses reveal the variation among all these attributes for the sites belonging to a particular cluster. Alternatively, the conserved interactions could be used as the basis for engineering any novel protein with specific ligand interaction. A recent study (38) reported the computational design of digoxigenin binding sites on protein scaffolds, thus implying the suitability of computational approaches for such applications.

## Conclusion

In conclusion, the PLIC database provides clusters of binding sites along with information about similarity among members within a cluster, common interactions in the cluster through alignments of sites, protein–ligand contacts, binding site environment properties and binding energetics that help in analyzing various attributes of protein–ligand interactions. This analysis is expected to be relevant in a number of ways, for understanding determinants of recognition, identifying critical residues responsible for binding of a given ligand, deriving sequence–structure–function relationships for evolutionary studies, as well as toward quantitative structure–activity relationship models, thereby helping in the process of drug design.

## Supplementary Data

Supplementary data are available at *Database* Online.

## Funding

Funding for open access charge: We gratefully acknowledge support from the Department of Biotechnology (DBT), Government of India, for financial support.

*Conflict of interest*. None declared.
